# Tailored Branched Polymer–Protein Bioconjugates for Tunable Sieving Performance

**DOI:** 10.1021/acsmacrolett.4c00059

**Published:** 2024-04-04

**Authors:** Kriti Kapil, Hironobu Murata, Grzegorz Szczepaniak, Alan J. Russell, Krzysztof Matyjaszewski

**Affiliations:** † Department of Chemistry, Carnegie Mellon University, 4400 Fifth Avenue, Pittsburgh, Pennsylvania 15213, United States; ‡ Faculty of Chemistry, University of Warsaw, Pasteura 1, 02-093 Warsaw, Poland; § Amgen Research, 1 Amgen Center Drive, Thousand Oaks, California 91320, United States

## Abstract

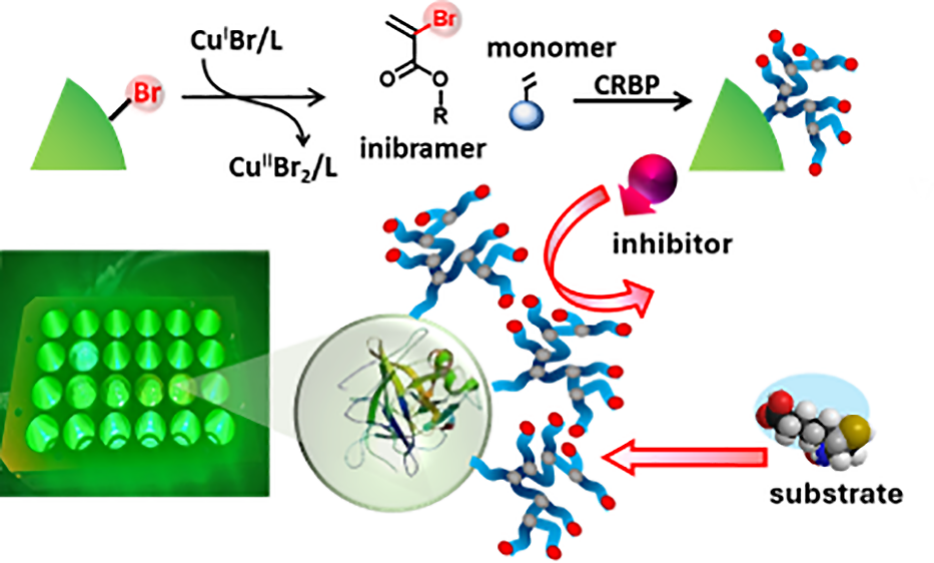

Protein–polymer conjugates combine the unique properties of both proteins and synthetic polymers, making them important materials for biomedical applications. In this work, we synthesized and characterized protein-branched polymer bioconjugates that were precisely designed to retain protein functionality while preventing unwanted interactions. Using chymotrypsin as a model protein, we employed a controlled radical branching polymerization (CRBP) technique utilizing a water-soluble inibramer, sodium 2-bromoacrylate. The green-light-induced atom transfer radical polymerization (ATRP) enabled the grafting of branched polymers directly from the protein surface in the open air. The resulting bioconjugates exhibited a predetermined molecular weight, well-defined architecture, and high branching density. Conformational analysis by SEC-MALS validated the controlled grafting of branched polymers. Furthermore, enzymatic assays revealed that densely grafted polymers prevented protein inhibitor penetration, and the resulting conjugates retained up to 90% of their enzymatic activity. This study demonstrates a promising strategy for designing protein–polymer bioconjugates with tunable sieving behavior, opening avenues for applications in drug delivery and biotechnology.

## Introduction

Protein–polymer conjugates play a crucial role in a wide range of biomedical and biotechnological applications. These hybrid materials combine the unique biological activities of proteins or enzymes with the advantageous chemical and physical properties of synthetic polymers.^1−3^ For example, attaching polymers to the surface of proteins can preserve or enhance their enzymatic activity even under harsh conditions, prolong circulation time by reducing renal clearance, protect proteins from antibodies and digestive enzymes, and make them responsive to factors such as pH, light, and temperature.^4,5^ Advanced synthetic techniques such as reversible deactivation radical polymerization (RDRP)^6−11^ and “click” chemistry have been instrumental in obtaining well-defined bioconjugates.^12−15^ In turn, the development of new analytical methods has improved the ability to determine the chemical structure and physical properties of bioconjugates.^16^ As a result, the synthesis of precisely engineered functional polymer bioconjugates has emerged as one of the central focuses in macromolecular engineering.^17−20^

The traditional “grafting-to” strategy has long been used to attach presynthesized polymers like poly(ethylene glycol) (PEG) to proteins, reducing immunogenicity by masking antibody binding sites.^21,22^ However, PEGylation strongly inhibits the cellular absorption and escape from endosomes, leading to a reduction in the effectiveness of the delivery system.^3,23,24^ In contrast, the “grafting-from” approach involves growing polymers directly from the protein surface. Atom transfer radical polymerization (ATRP) has been widely used for grafting-from proteins, enabling high grafting density, site-specific polymer growth, and the rational synthesis of protein–polymer conjugates with improved solubility, stability, and functionality.^25−29^

Despite these advances, simple protein–polymer conjugates do not fully retain their functionality, because the polymer chains do not completely eliminate interactions between the protein surface and other biomacromolecules. In the context of therapeutic enzyme-polymer conjugates, it is desirable to repel protein-antibody interactions and protease-mediated hydrolysis while retaining their activity toward substrates and ligands.

In 2012, the term “molecular sieving” was introduced to describe the polymer-mediated shielding of binding sites, which affects the permeation rates of ligands to the protein surface.^30^ Comb-shaped poly(oligo(ethylene glycol) methacrylate) (pOEOMA) polymers, when grafted from a chymotrypsin surface, created a molecular sieving effect by blocking larger macromolecules. Recently, we conducted a systematic analysis of bioconjugates synthesized from proteins functionalized with single- and double-headed ATRP initiators and observed a slower rate of diffusion and binding of ligands to the active site of the protein as a function of polymer grafting density.^31^ Subsequently, we investigated linear, branched, and comb-shaped architectures grown from the protein surface by ATRP and demonstrated that the extent of molecular sieving depends on the polymer grafting density.^32^

To enhance the biological activity of poly(glycerol)–protein conjugates, an optimal combination of composition, molecular weight, and polymer architecture was crucial.^33,34^ Densely grafted polymers have been studied, including the implantation of proteins in molecular bottlebrushes,^35^ the use of click chemistry to conjugate globular dendrimers,^36−39^ and grafting PEG macromonomers via ATRP to mask proteins.^40^ Hyperbranched polymers possess unique properties such as weak entanglement, low viscosity, and a globular conformation, making them excellent candidates for grafting from the surface of proteins.^41^ However, the lack of mild controlled radical polymerization techniques has limited progress in the field of branched polymer–protein bioconjugates.

Recently, we developed a fully oxygen-tolerant controlled radical branching polymerization (CRBP) technique in water using inibramer chemistry and dual photo redox/copper catalysis.^41^ The term “inibramer” refers to a monomer that can initiate the branching process only after it is incorporated into the polymer chain.^42,43^ A water-soluble inibramer, sodium 2-bromoacrylate (SBA), triggered branching during photoinduced ATRP of methacrylate monomers in one pot. As a result, well-defined branched polymers with controlled molecular weights, degrees of branching, and low dispersity values were obtained in water. The technique was extended to the grafting of well-controlled hyperbranched polymers directly from biomacromolecules.^41^

**Figure 1:**
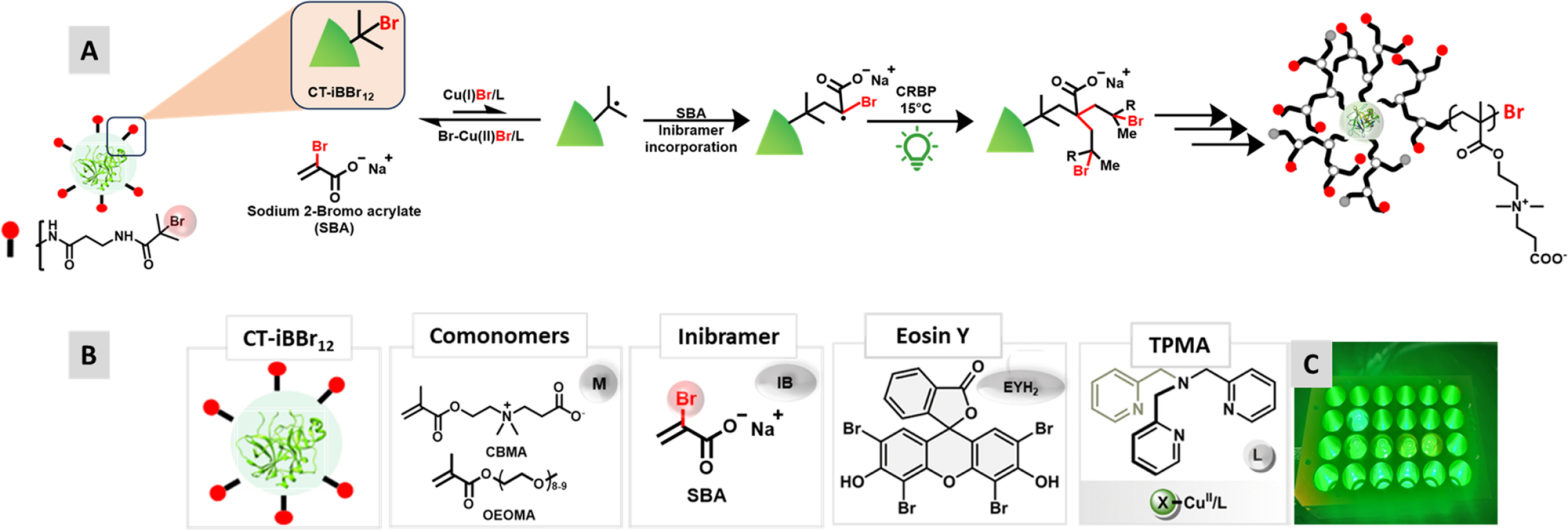
(A) Proposed mechanism for grafting-from CRBP using chymotrypsin macroinitiator (CT-iBBr_12_) to achieve tunable degree of branching. (B) Components of copolymerization in open-air CRBP (C) 24-well LED array setup irradiated with green light (λ = 525 nm, 50 mW/cm^2^).

Herein, using green-light-induced CRBP,^26,44,45^ and a high-throughput synthetic setup, we developed a first straightforward approach to introduce branching into protein–polymer hybrids using inibramers. We prepared well-defined bioconjugates of proteins with branched polymers allowing for tunable degrees of branching in one-pot (Figure 1). Subsequently we investigated and compared the sieving behaviors of synthesized bioconjugates.

## Synthesis and Characterization of CT-Branched Polymer Conjugates

Chymotrypsin (CT) is a digestive proteolytic enzyme that is widely used in enzyme replacement therapies to treat pancreatic insufficiency. CT was selected as a model protein because it is a well-studied proteolytic enzyme with a wide variety of commercially available inhibitors and substrates. Chymotrypsin macroinitiator (CT-iBBr_12_) with 12 -Br initiating sites per CT was synthesized according to a previously reported method.^46^ Sodium 2-bromoacrylate (SBA) inibramer stock solution was prepared by dissolving 2-bromoacrylic acid (BAA) in an aqueous solution of Na_2_CO_3_. The green-light-induced CRBP was carried out in parallel in 2 mL open vials placed on a 24-well LED array, which allowed reproducible light intensity (525 nm, 50 mW cm^–2^), irradiation time of 30 min, and low temperature (15−18 °C) during the polymerization reaction (Figure S1). Eosin Y (EYH_2_) was used as the photoredox catalyst, and CuBr_2_/TPMA (TPMA: tris(2-pyridylmethyl) amine) as the deactivator (Table 1). Phosphate-buffered saline (PBS) was used as the reaction medium to provide biocompatible conditions for chymotrypsin while suppressing dissociation of the [X–Cu^II^/L]^+^ deactivator, and to form the highly photoactive form of eosin Y (EY).^47^ Polymerizations were performed with varying ratios of SBA inibramer (2–20%) to obtain CT-hyperbranched polymer conjugates with a tunable degree of branching (Figure S2). Initial studies started with polymerization of the zwitterionic monomer, 3-[2-(methacryloyloxy) ethyl] dimethylammonium] propionate (CBMA) (Entries 2–7, Table 1). Additionally, oligo(ethylene oxide) methyl ether methacrylate (average *M*_n_ = 500, OEOMA_500_) was copolymerized with and without SBA inibramer to synthesize bioconjugates with a comb-like polymer backbone (entries 8 and 9, Table 1).

**Table 1:** Synthesis of Hyperbranched Polymer-Protein Bioconjugates via CRBP^a^

Entry	Sample Name	[M]/[SBA]/[I]	α_M_^b^ (%)	α_IB_^b^ (%)	*M*_n,th_^c^	*M*_n,abs_^d^	*Đ*
1.	CT-L- pCBMA	200/0/1	68	-	363 000	378 000	1.48
2.	CT-B-2%-pCBMA	200/2/1	78	84	374 000	301 000	1.65
3.	CT-B-4%-pCBMA	200/4/1	72	88	390 000	359 000	1.62
4.	CT-B-6%-pCBMA	200/12/1	70	85	385 000	352 000	1.40
5.	CT-B-10%-pCBMA	200/20/1	76	89	418 000	458 000	1.60
6.	CT-B-15%-pCBMA	200/30/1	78	88	450 000	556 000	1.57
7	CT-B-20%-pCBMA	200/40/1	75	87	416 000	588 000	1.52
8.	CT-L-pOEOMA	200/0/1	42	-	504 000	561 000	1.46
9.	CT-B-6%-pOEOMA	200/12/1	38	66	456 000	526 000	1.56

a Reaction conditions: [M]/[SBA]/[I]/[EYH_2_]/[CuBr_2_]/[TPMA]: 200/x/1/0.01/0.2/0.6, [M] = 300 mM, [SBA] = 6–60 mM, [I] = 1.5 mM ([CT-iBBr_12_] = 0.125 mM, each CT has 12 ATRP initiating sites) in 1X PBS, irradiated for 30 min under green light LEDs (527 nm, 50 mW cm^–2^), at 15 °C −18 °C. Reaction volume 2.0 mL, stirring at 300 rpm.

b Monomer and inibramer conversion was determined by using ^1^H NMR spectroscopy.

c Theoretical molecular weight (*M*_n,th_) was calculated based on conversion (i.e., *M*_n,th_ = [[M]/[I]] × MW_[M]_ × α_[M]_ + [SBA]/[I]] × MW_[SBA]_ × α_[SBA]_ + MW_[CT-iBBr12]_).

d Absolute molecular weight (*M*_*n, abs*_) determined by SEC in 1× DPBS coupled with multiangle light scattering detectors (MALS).

CT-hyperbranched polymer bioconjugates were then purified by dialysis in DI water and then lyophilized to obtain pure and dry CT-bioconjugates. The successful synthesis of branched methacrylate-based bioconjugates was demonstrated by SEC equipped with a multiangle light scattering (MALS) detector. Monomodal SEC traces were observed for the synthesized bioconjugates (Figure S3), with predictable absolute molecular weight value (*M*_n,abs_) (Table 1, entry 1–4). Using ≥10 mol % inibramer ratio caused gelation during the copolymerization (Table 1, entries 5–7) which led to a ≈ 40% variance between *M*_n,abs_ and *M*_n,th._ This inaccuracy in measurement was ascribed to gel formation observed during the synthesis of protein bioconjugates due to excessive radical generation. Furthermore, the bioconjugates exhibited a slightly broader molecular weight distribution, arising from the distribution of branching junctions during the copolymerization, a characteristic feature for copolymers with inibramers.^48,49^

**Figure 2:**
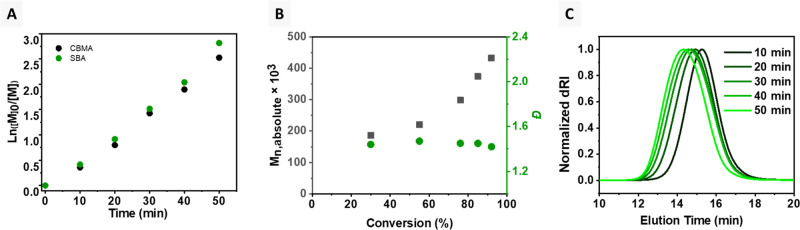
Copolymerization kinetics for grafting-from CRBP using molar ratios [CBMA]/[SBA]/[CT-iBBr_12_]/[EYH_2_]/[CuBr_2_]/[TPMA] = 200/12/0.08/0.01/0.2/0.6 at 15 °C −18 °C under green light LEDs (527 nm, 50 mW cm^–2^). (A) First-order kinetic plot, (B) evolution of molecular weight and molecular weight distribution with conversion, and (C) SEC traces evolution with time.

The copolymerization kinetics were studied to determine the relative rate of incorporation of the inibramer into the branched copolymer grafts. The EY/Cu-catalyzed CRBP was performed using molar ratios of [CBMA]/[SBA]/[CT-iBBr_12_]/[EYH_2_]/[CuBr_2_]/[TPMA] = 200/12/0.08/0.01/0.2/0.6. The copolymerization exhibited first-order kinetics with a short induction period of 5 min, followed by a rapid polymerization, reaching 85% CBMA and 87% SBA monomer conversion after 50 min (Figure 2A). The polymerization kinetics revealed random incorporation of the SBA inibramer, resulting in uniform distribution of branching junctions along the polymer backbone. The molecular weight of the CT-hyperbranched polymer bioconjugates increased as a function of monomer conversion, and the dispersity values remained relatively low (*Đ* ≤ 1.5) during the CRBP (Figure 2B,C).

To analyze the polymer architecture, the conformation plots were generated for both linear and branched polymer grafts (see the Supporting Information in Figure S5). These plots illustrate the correlation between the polymer size or its intrinsic viscosity and molar mass. The slope, expressed as d log(r_g_)/d log(M), provides insights into the conformation of the polymer, whether it resembles a sphere, a random-coil or rod-like. The presence of branching was determined by analyzing the slopes in these conformation plots, utilizing analogous linear polymers as a reference. To facilitate the cleavage of the grafted polymer chains from the CT, microwave-assisted acid hydrolysis was performed (Figure S4).^50−52^ Following this process, we subjected the cleaved polymers to thorough purification by dialysis. We then performed a comprehensive characterization approach using techniques such as ^1^H NMR and SEC-MALS equipped with triple detectors, an inline viscometer, and dynamic light scattering (DLS) (Table S1). The results revealed that the absolute molecular weights of both linear and branched polymers were close to their theoretical values, demonstrating a narrow molecular weight distribution, with branched polymers having slightly higher *Đ* values than their linear counterparts. Additionally, the relationship between the root-mean-square (RMS) radius, intrinsic viscosity, and molecular weight, showed that branched polymers exhibited lower slope values compared to their linear counterparts. This difference indicated successful branching in the grafted polymers and that the branched polymers had a smaller hydrodynamic radius (Figure S5).

### Enzymatic Activity of the Synthesized CT-Branched Polymer Bioconjugates

After confirming the hyperbranched structure of the bioconjugates, we determined their enzymatic activities using the common small peptide substrates *N*-suc-l-Ala-l-Ala-l-Pro-l-Phe-pNA (suc-AAPF-pNA) (Table 2). The Michaelis–Menten kinetic parameters of native CT and linear and branched polymer CT conjugates were estimated to compare their enzymatic activities as well as the shielding effects of modified polymers (see Supporting Information). Compared with native CT, the *K*_M_ values of linear and branched pCBMA CT conjugates were reduced by ∼20–30%. i.e., increased affinity for small peptide substrates, as reported in previous studies of pCBMA-modified CT conjugates.^24^ The turnover (*k*_cat_) of the conjugates was also reduced by about 35–75%, which is different from the previously reported results for conjugates prepared by the activators regenerated by electron transfer (ARGET) ATRP method, where *k*_cat_ did not change significantly after conjugation with pCBMA.^24^ To investigate the cause of decreased enzymatic activity, native CT in a polymerization solution containing eosin Y was irradiated with green light (527 nm, 50 mW cm^–2^), and its enzyme activity was estimated (see Supporting Information). Native CT in 1× PBS and 10% DMSO, irradiated under green light for 60 min showed approximately 35% reduction in enzymatic efficiency compared to the control. Native CT irradiated with green light in the presence of eosin Y showed 80% reduction in enzymatic activity compared to the control (entry 3, Table S2).^53^ Similar reduction in activity of Native CT was observed in the presence of ligand, copper, and 100 mM sodium pyruvate (entries 4–6, Table S2 and Figure S6). This demonstrates the damage to proteins due to prolonged exposure to light irradiation and highlights the importance of achieving rapid kinetics while maintaining control over the polymerization during photo-ATRP.^53^ The effect of lower energy light (red and NIR) should be investigated in future studies. CT-pCBMA conjugates synthesized under the same conditions showed comparable or slightly improved residual activity (entries 7–10, Table S2). Furthermore, both cases of linear and hyperbranched pOEOMA CT conjugates showed an increase in *K*_M_ and a decrease in *k*_cat_. The enzymatic efficiency of pOEOMA CT conjugates decreased due to green light irradiation in the presence of eosin Y and a decrease in affinity with the substrate due to the shrunken pOEOMA on the CT. The reduced activity in CT-pOEOMA conjugates was due to shielding of the enzyme active site by collapsed pOEOMA rather than to polymerization conditions, as previously reported.^27^

**Table 2:** Michaelis–Menten Parameters of the Native CT and the Linear and Hyperbranched Polymer CT Conjugates for suc-AAPF-pNA

	*K*_M_^a^	*V*_max_^a^	*k*_cat_^a^	*k*_cat_/*K*_M_^a^
Sample Name	(μM)	(μM s^–1^)	(s^–1^)	(μM^–1^ s^–1^)
CT	90.5 ± 10.7	1.68 ± 0.05	41.9 ± 1.2	0.462 ± 0.056
CT-L-pCBMA	64.0 ± 11.4	0.40 ± 0.02	10.1 ± 0.4	0.158 ± 0.029
CT-B-2%-pCBMA	70.4 ± 10.2	0.57 ± 0.02	14.3 ± 0.5	0.203 ± 0.030
CT-B-4%-pCBMA	63.4 ± 8.3	0.63 ± 0.02	15.7 ± 0.5	0.248 ± 0.033
CT-B-6%-pCBMA	75.4 ± 9.3	0.50 ± 0.02	12.4 ± 0.4	0.165 ± 0.021
CT-L-pOEOMA	144.5 ± 17.6	0.08 ± 0.01	2.1 ± 0.1	0.014 ± 0.002
CT-B-6%-pOEOMA	158.1 ± 25.0	0.07 ± 0.01	1.7 ± 0.1	0.011 ± 0.002

a Michaelis–Menten kinetic parameters were estimated at 37 °C for CT and conjugates with *N*-suc-AAPF-pNA. *K*_M_ and *V*_max_ were calculated using EnzFitter software. *k*_cat_ was calculated by dividing *V*_max_ by the initial enzyme concentration, [CT]_0_ = 40 nM.

### Study of Sieving Effect of Branched Polymers on the Conjugates

**Figure 3:**
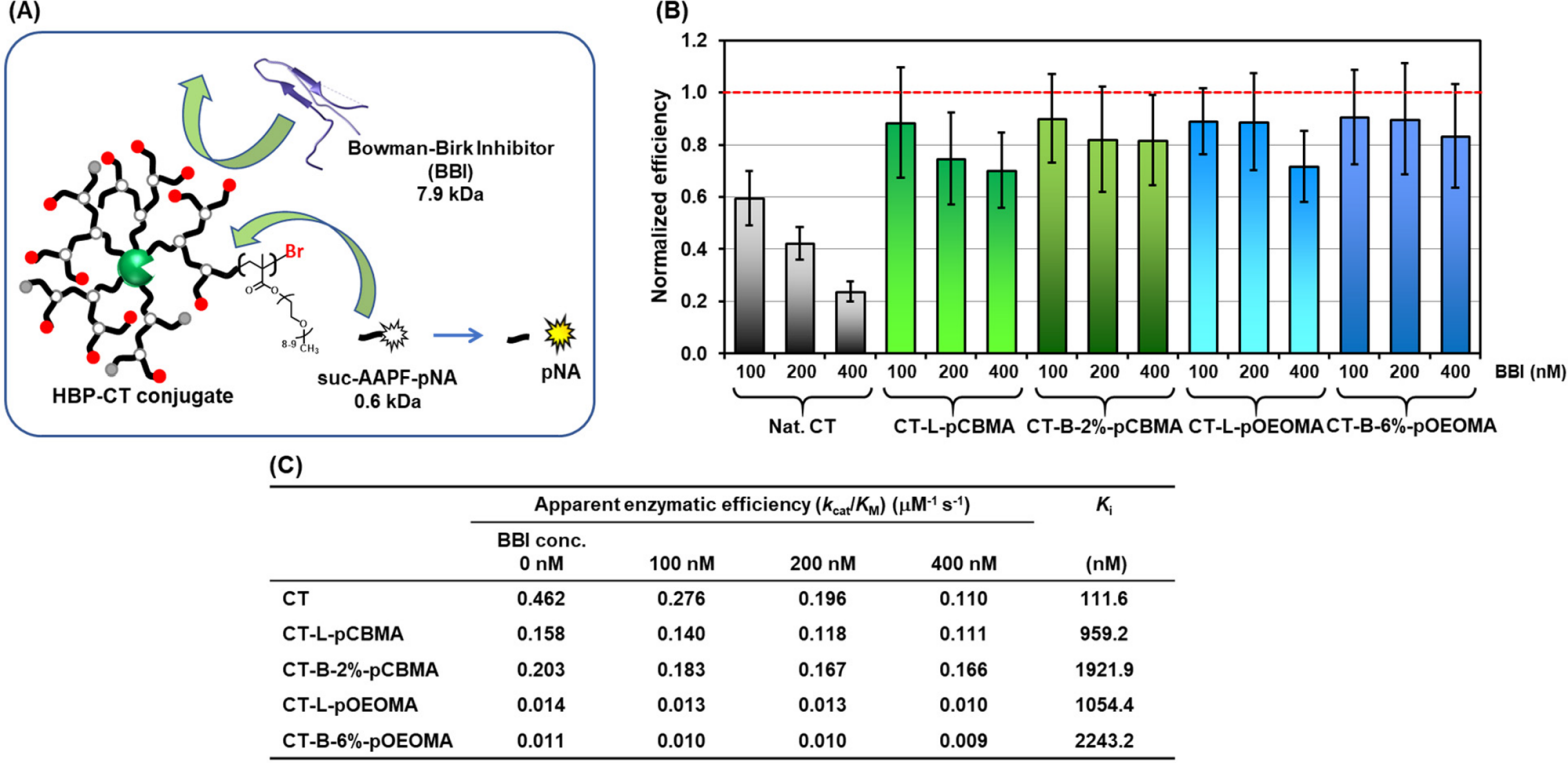
(A) Sieving effect of the hyperbranched polymer on the conjugates. (B) Normalized enzymatic efficiency of native chymotrypsin, CT-L, B-2%-pCBMA, CT-L-and B-6%-pOEOMA in the presence of 100–400 nM BBI. (C) Apparent enzymatic efficiency in the absence and presence of BBI of native chymotrypsin, CT-L, B-2%-pCBMA, CT-L-and B-6%-pOEOMA, and estimated inhibition constant toward BBI.

Linear polymer–protein conjugates are limited in grafting density because proteins have an inherently fixed number of functional amino acids, such as lysine, that can be modified. As a result, they cannot completely prevent protein–protein interactions. On the other hand, proteins densely coated with comb-shaped polymers offer a superior shielding effect, effectively preserving functionality while eliminating undesirable protein–protein interactions.^24,40^ To investigate the sieving effect on CT-branched polymer conjugates, we compared the activity of these conjugates toward the small peptide substrates in the presence of a protein inhibitor. An effective sieving effect on the branched polymer should impede the penetration of excess protein inhibitor, resulting in no significant change in the activity of the conjugate toward small substrates (Figure 3A).

The enzymatic efficiency of the CT-polymer conjugates was determined in the presence of a known competitive inhibitor of CT, the Bowman–Birk inhibitor (BBI). In the presence of BBI, the activity of native CT toward small substrates (suc-AAPF-pNA) was reduced by 75% when 10 equiv of BBI (400 nM) were added relative to native CT (40 nM) (Figure 3B, Tables S3 and S4). However, the CT-L-pCBMA conjugate was able to reduce BBI inhibition due to the sieving effect of the grafted pCBMA. This conjugate maintained its enzymatic activity for small peptide substrates at 70% compared with the activity without BBI incubation (Figure 3B). Furthermore, the CT-B-2%-pCBMA conjugate exhibited even greater resilience to BBI inhibition, maintaining over 80% activity in the presence of BBI. This suggests that the branched polymers interacted intimately due to the higher degree of branching, creating an effective sieving effect. The linear and branched-pOEOMA CT conjugate also exhibited resistance to BBI penetration into the CT active site. In the presence of BBI (400 nM), the linear and branched polymer conjugates retained 70% and 80% of their enzymatic activity, respectively (Figure 3B). This effect could be attributed to the relatively hydrophobic nature of pOEOMA, which collapsed onto the CT surface, enhancing the sieving effect. In Figures 3B and 3C, as the BBI inhibitor increases, the decrease in apparent enzymatic efficiency of the branched polymer conjugates was smaller compared to that of the linear polymer conjugates. The decreased activity of CT-B-4% and 6%-pCBMA in the presence of BBI was similar to that of the CT-L-pCBMA conjugate (Tables S3–S5). These observations suggest that the control over branching density and location, as well as the structure–activity relationship between the protein and polymer architecture, requires detailed consideration.

In Figure 3B, a clear trend of the shielding effect due to branching was confirmed, but the statistical significance of the effect is uncertain, because each error is large. To estimate the inhibition constant of BBI, the activity of the conjugate toward small peptide substrates in the presence of different BBI concentrations was measured and the respective apparent *K*_M_/*V*_max_ was plotted (Table S3–S5, Figure S7). The *K*_i_ of BBI relative to that of native CT was 111 nM (Figure 3C and Table S9). For CT-L or B-2%-pCBMA conjugates, the *K*_i_ values increased by 8–17 times, respectively, indicating effective shielding of the BBI by the grafted polymer. In particular, since there is a 2-fold difference in each *K*_i_, conjugate prepared by the branching strategy had a significant impact on their shielding effect. CT-L and B-6%-pOEOMA conjugates also showed 10 and 20-fold increase in their *K*_i_ values, respectively. As a control the *K*_i_ of BBI relative to native CT was evaluated for CT treated under the polymerization conditions (entry 5, Table S2), *k*_cat_ was significantly reduced (≈ 80%) by green light irradiation and the sample exhibited 4 times lower *K*_i_ value (29 nM) (Table S10, Figure S9). As with CT-L and B-2%-pCBMA, the shielding effect of branching on protein inhibitors was meaningfully demonstrated by observing the respective *K*_i_ values.

Additional inhibition experiments were performed using the small protein inhibitor AP (6.5 kDa) compared with BBI (average 8 kDa). We measured the activity of native CT and the conjugates toward small peptide substrates in the presence of different AP concentrations and estimated the inhibition constants of AP (Table S6–S9 and Figure S8). AP had a larger inhibition than BBI against native CT because it had a smaller *K*_i_ value. For CT-L or B-2%-pCBMA conjugates, the *K*_i_ values increase by 6–8 times, respectively, demonstrating the AP penetration is shielded by the grafted polymer. However, since the difference in each *K*_i_ was small compared to BBI, the shielding effect by branching decreased. For CT-L or B-6%-pOEOMA conjugates, both *K*_i_ values increased 10 times as compared with native CT. However, since there was almost no difference in the values, no improvement in shielding effect due to branching was observed. In the future, we plan to use protein inhibitors that are larger than BBI, such as alpha 1-antichymotrypsin (54 kDa), to investigate the shielding effect of their branching. It is known that the thermal and pH stability of dense polymer–protein conjugates is improved compared to native proteins.^54^ Therefore, we will continue to study the thermal and pH stability of conjugates prepared by a densely branched polymer conjugation strategy.

In summary, this study presents a promising approach for tailoring protein–polymer bioconjugates with a tunable sieving behavior. CT-branched polymer conjugates prepared by controlled radical branching polymerization using the inibramer retain the same or higher enzymatic activity as linear polymer conjugates and provide significant shielding effects and stabilization against protein inhibitors. The research endeavors will continue to expand, encompassing a broader range of target proteins and molecular weights of modifying polymers. We will continue to investigate how hyperbranched polymers affect the morphology and functionality of protein conjugates, further advancing our understanding of these intriguing interactions. We will also explore other photosensitizers for photoinduced CRBP that require a lower energy light (red or NIR). This method effectively protects proteins, as they can avoid threats from antibodies and digestive enzymes; we plan to apply this method to medical proteins such as uricase which is used to treat severe gout and asparaginase for the treatment of acute lymphoblastic leukemia.

## Supporting Information Available

Details about materials, instrumentation, experimental procedures and additional results and analysis included.
