# Supplementary material for: Tailored Branched Polymer–Protein Bioconjugates for Tunable Sieving Performance
Source: ACS Macro Lett. 2024 Apr 4;13(4):461–7. doi: 10.1021/acsmacrolett.4c00059 (PMC11025119; doi:10.1021/acsmacrolett.4c00059)
Supplement: Supplementary file 1 — mz4c00059_si_001.pdf [file mz4c00059_si_001.pdf]

Tailored Branched Polymer-Protein Bioconjugates for Tunable Sieving Performance

Kriti Kapil[1], Hironobu Murata[1], Grzegorz Szczepaniak[1,2], Alan Russell[3], Krzysztof Matyjaszewski [1,*]

[1] Department of Chemistry, Carnegie Mellon University, 4400 Fifth Avenue, Pittsburgh, PA 15213, United States.

Corresponding Author Email: km3b@andrew.cmu.edu, matyjaszewski@cmu.edu

[2] Faculty of Chemistry, University of Warsaw, Pasteura 1, 02-093 Warsaw, Poland.

[3] Amgen Research, 1 Amgen Center Drive, Thousand Oaks, California 91320, United States

Experimental Details

Materials

All chemicals were purchased from commercial sources and used as received unless otherwise noted. Tris(2-pyridylmethyl) amine (TPMA, 99%) was purchased from AmBeed. 2-Bromoacrylic acid (BAA, 95%) was purchased from Fischer Scientific. 2-Hydroxyethyl 2-bromoisobutyrate (HO-EBiB, 95%), copper (II) bromide ($CuBr_2$, 99.99%), sodium carbonate (Na_2CO_3, ≥99.0%), sodium phosphate dibasic (Na_2HPO_4, ≥99.0%), potassium phosphate monobasic (KH_2PO_4, ≥99.0%), and Eosin Y (EHY_2, 99%) were purchased from Sigma-Aldrich. The monomers oligo(ethylene oxide) methyl ether methacrylate, OEOMA500 and OEOMA300 were purchased from Sigma-Aldrich and passed through a column of basic alumina to remove inhibitor prior to use. 3-[[2-(Methacryloyloxy) ethyl] dimethylammonium] propionate (CBMA, 98%) was purchased from TCI. 10X PBS and 10X DPBS solutions ware purchased from Thermo Fisher Scientific. Water (HPLC grade), dimethyl sulfoxide (DMSO, ≥99.7%) and *N, N*-dimethylformamide (DMF, ACS grade) were purchased from Fisher Chemical. α-Chymotrypsin from bovine pancreas, Bowman-Birk trypsin-chymotrypsin inhibitor from Glycine max (soybean), and aprotinin from bovine lung were purchased from Sigma-Aldrich. Peptide substrate, *N*-Succinyl-L-Ala-L-Ala-L-Pro-L-Phe-*p*-nitroanilide (suc-AAPF-pNA), was purchased from Bachem (Torrance, CA).

Instrumentation

Nuclear Magnetic Resonance (NMR)

^{1}H NMR spectra were recorded on *Bruker* Avance III 500 MHz spectrometers with D_2O or DMSO-d6 used as the solvent.

Size Exclusion Chromatography with Multi-Angle Light Scattering (SEC-MALS- 1X DPBS eluent)

SEC-MALS measurements of bioconjugates were performed using Agilent SEC system (*Agilent*, 1260 Infinity II with UV detector) coupled with MALS, DLS, UV, Viscometer and RI detectors (*Wyatt Technology*, USA). Measurements were performed using *Waters* Ultra hydrogel Linear column with 1X DPBS as an eluent at room temperature and the flow rate of 0.5 mL/min.

Chymotrypsin-Macroinitiator (CT-iBBr$_{12}$) Synthesis:

Chymotrypsin (CT) was functionalized with ATRP initiators by covalent modification of the lysine groups of CT with NHS-iBBr following the previously reported protocol. [1] CT (1.0 g) was dissolved in 100 mM sodium phosphate buffer (pH = 8.0) at 0 °C. *N*-2-bromo-2-methylpropanoyl-β-alanine *N*′-oxysuccinimide ester (1.0 g, 3.0 mmol) dissolved in 1 mL of DMSO was then added dropwise. The mixture was stirred in a refrigerator for 4 h at 4 °C. The modified protein-macroinitiator was then dialyzed against 1X PBS for 48 hours at 4 °C using a 10 kDa molecular weight cut-off membrane, changing the dialysis buffer eight times. The dialyzed solution was lyophilized and stored at -20 °C. The number of lysines substituted with ATRP initiators (12 initiators/CT) was determined by fluorescence spectroscopy with a fluorescamine assay, using a calibration curve of the native protein. The molecular mass of CT-iBBr$_{12}$ was estimated to 28.2 kDA

High-throughput set-up for CT-Polymer Bioconjugate Synthesis

Polymerizations were carried out in open-to-air vials under 24-well green LEDs equipped with Lumidox II controller, Thermal Transfer Deck, and Para-dox Aluminum Reaction Block (Analytical Sales and Services, Inc., Flanders, NJ) (see Figure S1).

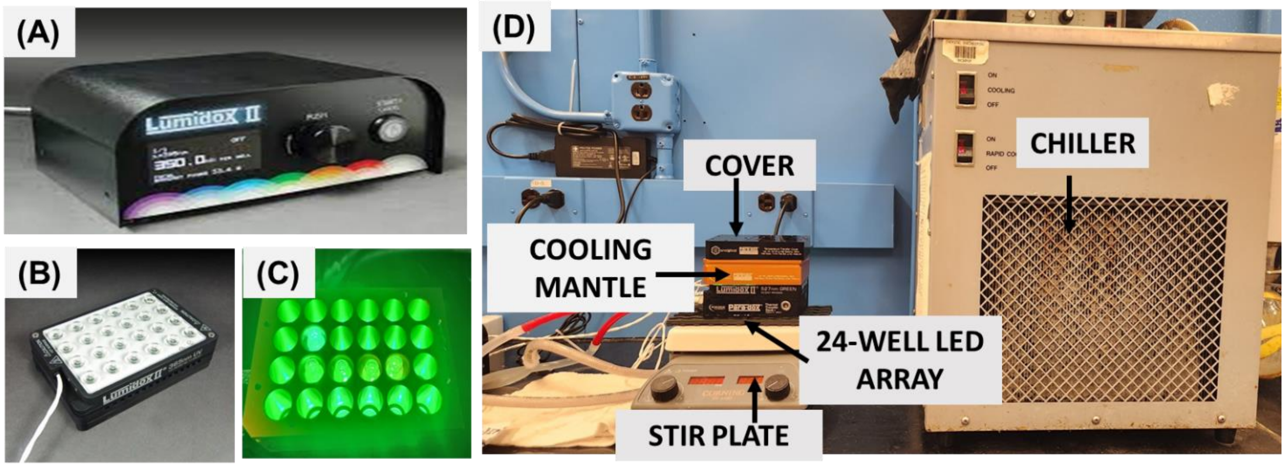

Figure S1. (A) Lumidox II controller (B) 24-well LED array (C) irradiated with green light (λ=525nm, 50 mW/cm^2) (D) complete set-up for synthesis of bioconjugates by photo-ATRP in open-air

Statistics

Data from at least two independent experiments are reported as mean ± standard deviation (SD).

Procedures

General procedure for synthesis of hyperbranched polymer-protein bioconjugates by CRBP.

Prior to polymerizations, stock solutions of $CuBr_2$ (33.5 mg in 20.0 mL of DMSO), TPMA (13.06 mg in 1.0 mL of DMSO), and EYH_2 (0.97 mg in 1.0 mL of DMSO) were prepared. The stock solution of SBA (sodium 2-bromo acrylate) was prepared by dissolving 2-bromoacrylic acid (BAA, 136 mg) in 5 mL of HPLC grade water consisting of 42.4 mg of Na_2CO_3.

In a 2 mL volumetric flask, CT-$iBBr_{12}$ (7.0 mg) and 1.5 mmol of monomer, CBMA- 137.5 mg (Table 1, entry 1-7) or $OEOMA_{500}$- 300mg (Table 1, entry 8-9) was weighed, $CuBr_2$ stock (80 μL), TPMA stock (40 μL), EYH_2 stock (20 μL), DMF (20 μL) as internal standard) and 10X PBS solution (200 μL) were then added. The ratio of SBA inibramer was varied to achieve desired degree of branching from 2-20% (Figure S2). Finally, HPLC grade water was added to the mark on the volumetric flask, and the reaction mixture was stirred on a vortex. The final concentrations were CBMA/$OEOMA_{500}$ (300 mM), CT-$iBBr_{12}$ (0.125 mM) leading to [-Br] = (1.5 mM), EYH_2 (15 μM), $CuBr_2$ (0.3 mM), TPMA (0.9 mM), DMSO (10% v/v). Then the ATRP cocktail was added transferred to 2 mL Agilent autosampler vial equipped with a narrow magnetic stirrer. The polymerization mixture was stirred at 300 rpm for 30 min under green LEDs (525 nm, 50 mW/cm^2) using high-throughput set-up equipped with cooling as shown in Figure S1.

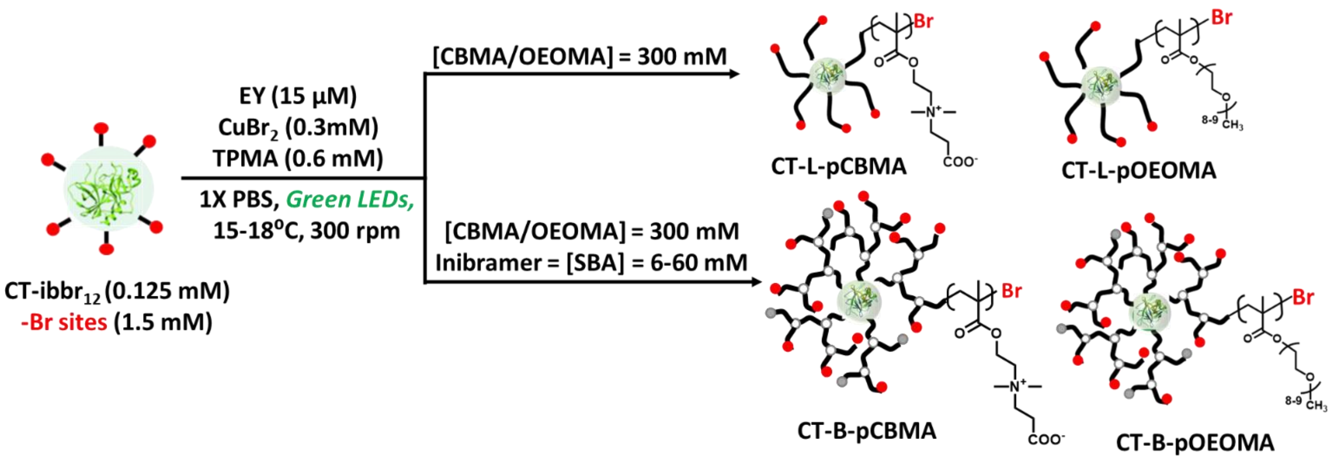

Figure S2. Scheme for synthesis of chymotrypsin bioconjugates with linear and branched pCBMA and pOEOMA.

Purification and characterization

During photo-ATRP the samples were taken and analyzed by ^{1}H NMR to compute monomer conversion and theoretical molecular weight of the synthesized bioconjugates using the equation $M_{n,th}=[M/I]\times MW_M\times\alpha M+[SBA/I]\times MW_{SBA}\times\alpha SBA+MW_{CT\text{-}iBBr12}$ (Table 1).

Post-polymerization the protein-polymer bioconjugate was purified by dialysis at 4^{0}C using 30k DA molecular weight cut-off membrane for 48 h in 1X PBS. The dialyzed solution was lyophilized and analyzed by SEC-MALS using 1X DPBS as the eluent.

The SEC-traces of the bioconjugates revealed monomodal traces with molecular weight higher than CT-iBBr$_{12}$. The measured absolute molecular wight agreed well with the theoretical molecular weight indicating well-controlled grafting from CT-surface. (Figure S2).

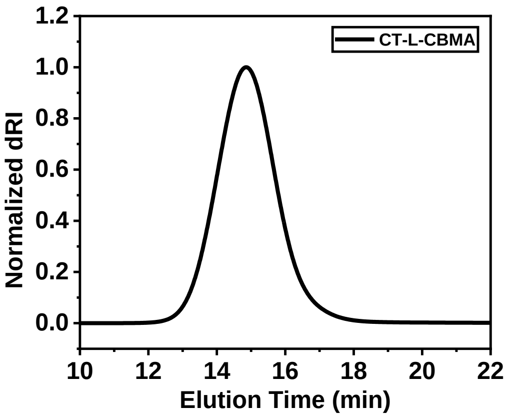
CT-L-CBMA
Normalized dRI
Elution Time (min)

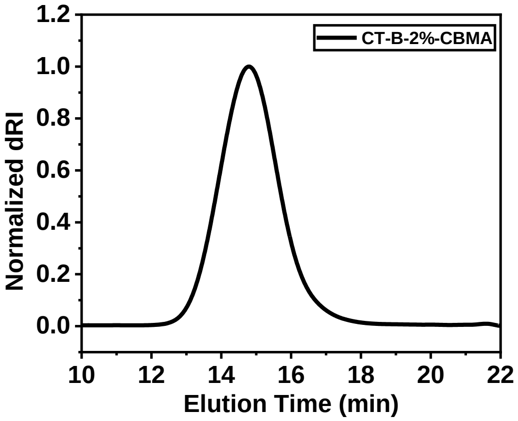
CT-B-2%-CBMA
Normalized dRI
Elution Time (min)

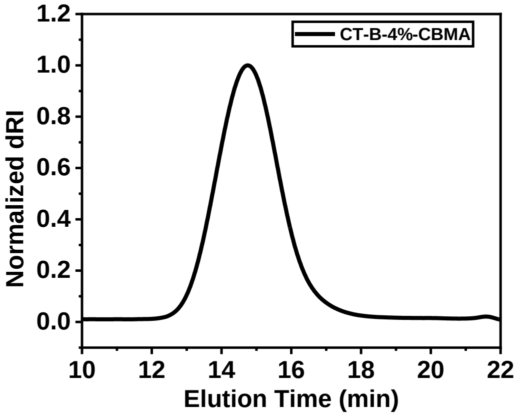
CT-B-4%-CBMA
Normalized dRI
Elution Time (min)

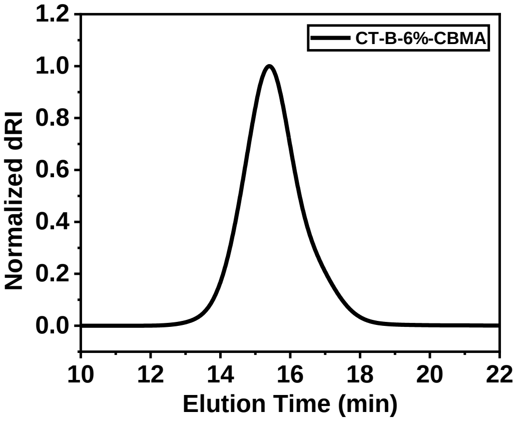
CT-B-6%-CBMA
Normalized dRI
Elution Time (min)

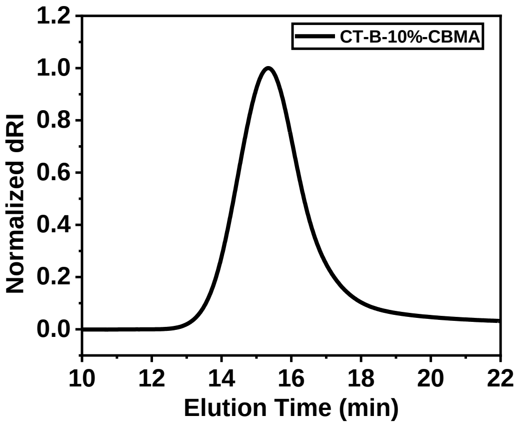
CT-B-10%-CBMA
Normalized dRI
Elution Time (min)

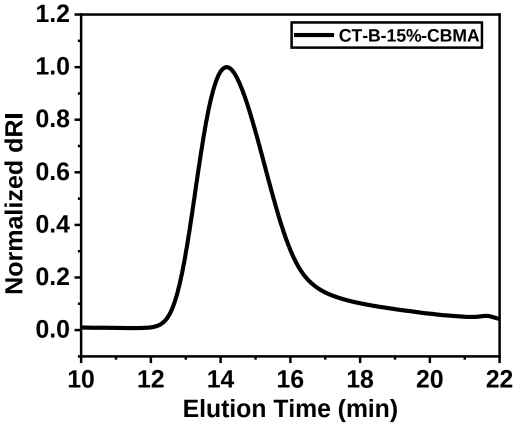
CT-B-15%-CBMA
Normalized dRI
Elution Time (min)

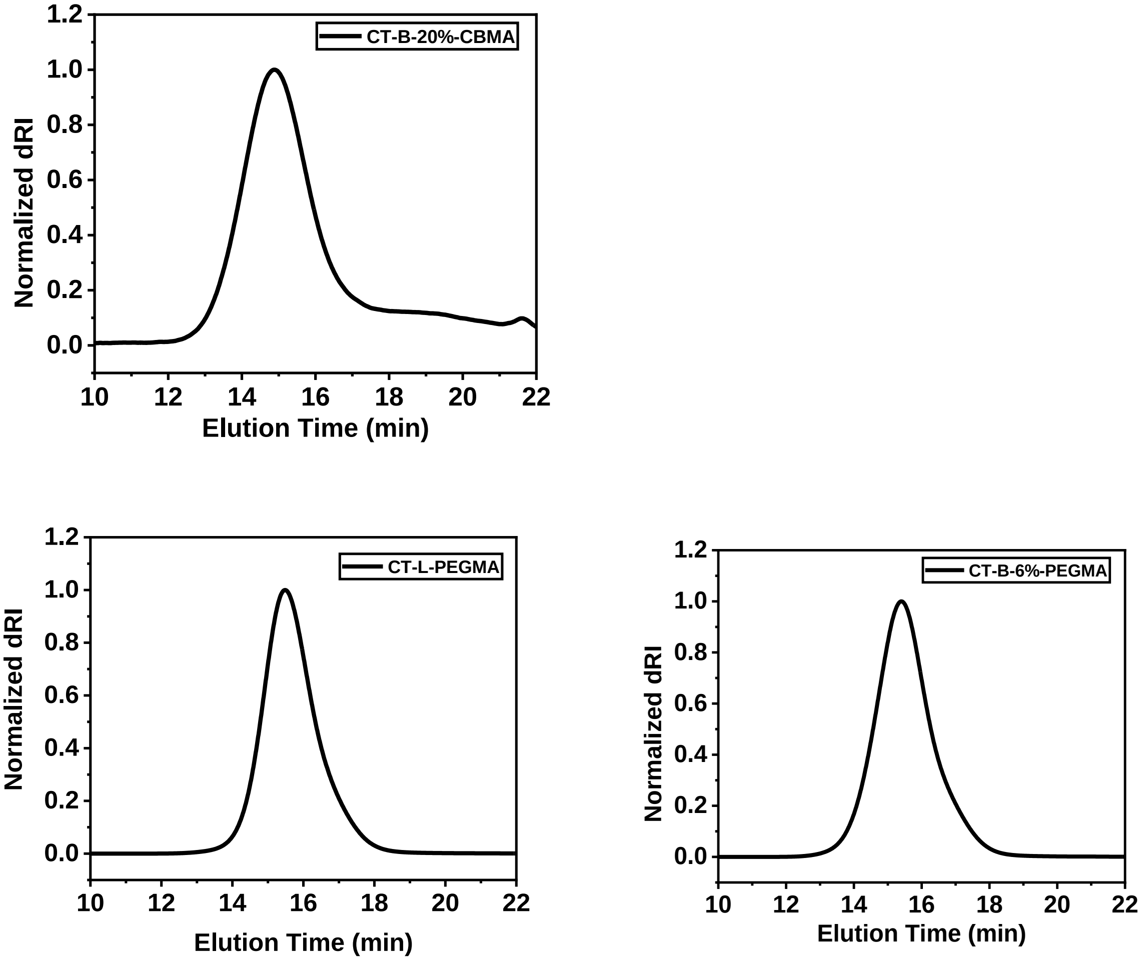

Figure S3. SEC-MALS traces of CT-polymer bioconjugates from Table 1.

Kinetics of copolymerization for synthesis of CT-branched polymer bioconjugate (Figure 2)

The copolymerization kinetics during the grafting-from CRBP was carried out by preparing the ATRP "cocktail" (2 mL) according to the general procedure. The final concentrations were CBMA (300 mM), SBA (18 mM) CT-iBBr$_{12}$ (1.5 mM), EYH$_2$ (15 μM), CuBr$_2$ (0.3 mM), TPMA (0.9 mM), DMSO (10% v/v). The polymerization mixture was added to a 2 mL Agilent autosampler vial equipped with narrow magnetic stirrer and stirred at 300 rpm for 50 min under 24-well green LEDs array (525 nm, 50 mW/cm^2). Samples were drawn at various time intervals to monitor by ^{1}H NMR (D$_2$O), the diminishing peaks corresponding to [CBMA] and [SBA] vs. time.

Microwave assisted acid hydrolysis for cleavage of grafted polymer from CT-polymer hybrids.

One of the important characterizations of polymer-protein hybrids is the estimation of total molar mass and its dispersity, for instance, it allows the calculation of the protein-to-polymer weight ratio of the protein-polymer hybrid (PPH) molecule. The development of SEC-MALS has made it possible to easily estimate the total molecular weight of PPH and its dispersity. Characterization of individual grafted polymer chains on PPH is also important for evaluating polymerization behavior and investigating its architecture. Previously, the polymer chains were cleaved from the PPH by base or acid hydrolysis to characterize the polymer and estimate the total molar mass of the PPH.[2-6]

Basic hydrolysis was performed with high concentrations of sodium hydroxide to cleave the polymer from PPH using an ATRP initiator with an ester group. This method can cleave the polymer chain from PPH in a short time, but since the protein is not completely hydrolyzed, it is necessary to extract the polymer with an organic solvent and isolate it for further characterization. Acidic hydrolysis, which is used for proteolysis, can hydrolyze the amide bond, and thus release the grafted polymer from PPH using an amide-linked ATRP initiator. Standard acidic hydrolysis of proteins requires a long reaction time of 24-48 hours at 110°C in 6N aqueous hydrochloric acid. Since proteins are hydrolyzed into small amino acids and oligopeptides, cleaved polymers can be easily isolated by dialysis or centrifugal filters.

Microwave-assisted acid hydrolysis (MAAH) is a greener route which uses a sealed reaction vessel enabling hydrolysis at high reaction temperatures and can greatly shorten the reaction time and therefore the energy expenditure. Here, MAAH was performed to cleave the polymer chains from the branched CT conjugates. Four CT-polymer bioconjugates consisting of linear POEOMA/PCBMA and branched POEOMA/PCBMA containing 6 mol% of SBA were analyzed.

4M $MeSO_3H$, which has a high boiling point was used instead of hydrochloric acid to avoid evaporation during hydrolysis. MAAH was performed at 160 °C for 45 minutes. After the solution returned to room temperature, the cleaved polymers were purified by dialysis and then lyophilized. The chemical structures of the obtained polymers were confirmed by ^1H NMR (Figure S4). After acid hydrolysis, the integrated value of methyl ether protons (3.3 ppm) in the polymer chain decreased by about 40% compared to the methyl protons in the polymer backbone. Since no decrease in methylene protons (around 4.2 ppm) adjacent to esters in the polymer chain was observed, some methyl ethers and ethylene glycol were considered to have desorbed. Similarly, cleaved linear and branched PCBMA polymer backbones were also studied by ^1H NMR (Figure S4). Interestingly, after MAAH of CT-L or B-PCBMA, the propionic acid moiety of the PCBMA side chain was completely detached and replaced with MSA, but the main chain structure was not affected, so samples were continued to be analyzed for branching.

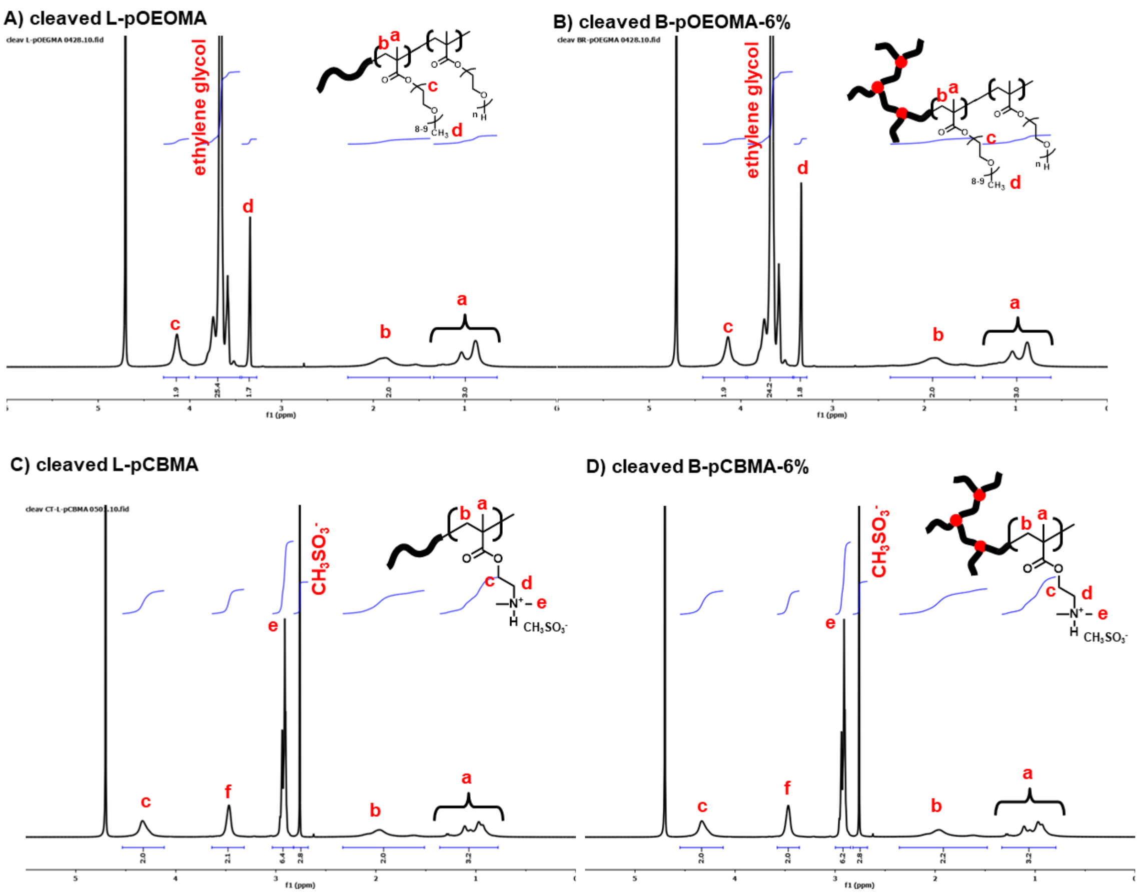

Figure S4. ^{1}H NMR spectra of CT-polymer conjugates after MAAH. A) cleaved p(OEOMA) from CT-L-pOEOMA, B) cleaved branched pOEOMA from CT-B-pOEOMA, C) cleaved pCBMA from CT-L-pCBMA and D) cleaved branched pCBMA by MAAH from CT-B pCBMA in D_2O, respectively.

Method:

The synthesis of PPH with linear POEOMA/PCBMA and branched POEOMA/PCBMA containing 6 mol% of SBA was carried out using optimized conditions, by irradiating the ATRP reaction mixture for 50 min under green light LEDs (λmax = 527 nm, 50 mW cm^{-2}) 15^{0}C -18^{0}C. It should be noted that a slightly longer irradiation time as compared to Table 1, was used to achieve a higher conversion of monomers as analyzed by ^{1}H NMR (Table S1).

20 mg of PPH synthesized above, was dissolved in 2mL of 4M methanesulfonic acid. The sample was transferred to a sealed vial and the reaction was carried out at 160 °C for 45 minutes using the Microwave reactor (Monowave 200, Anton Paar, Ashland, VA). After the sample had returned to room temperature, the solution was dialyzed in deionized water using a MWCO 3.5 kDa dialysis membrane for 24 hours. After lyophilization, samples were characterized by ^{1}H NMR and SEC-MALS (Table S1). The $M_{n,th}$ in Table S1 indicates the

computed theoretical molecular weight for each of the 12 polymer backbones grafted from the CT surface.

Characterization of cleaved polymer samples:

The cleaved polymer samples were then characterized by SEC-MALS equipped with triple detectors (refractive index (RI), ultraviolet (UV), light scattering (LS)) together with an inline viscometer and dynamic light scattering (DLS) detector to evaluate rms radius and intrinsic viscosity of the polymer samples **(Table S1).**

To identify and characterize branching, information about the molar mass and the molecular size is pertinent. MALS provides both pieces of information simultaneously and independently. The MALS detector is connected to an analytical separation technique SEC to determine branching parameters as a function of molar mass. Branching can be easily identified from the slopes of the conformation plots of all linear and branched polymers in thermodynamically good solvents. The parameters of interest are typically the branching ratio and the number of branch units per molecule. According to Zimm and Stockmayer equation, the 'branching ratio', g

$$g = (R^2_{\text{branched}} / R^2_{\text{linear}})_M$$

where R^2_x is the mean square radius of branched and linear polymers having the same molar mass M.

MALS has a limitation in determining RMS radius for relatively small polymers with Rg < ≈ 10 nm, which corresponds to a molar mass of ≈ 10^5 g/mol. In the case of such polymers, an alternative size parameter such as intrinsic viscosity and SEC elution volume were used.

Table S1: Characterization of cleaved polymer samples by MAAH of CT-linear and branched POEOMA/PCBMA

Entry	Sample Name (Cleaved)	[M]/[SBA]/[I]	M(%)	$^a\alpha_{IB}$ (%)	$^bM_{n,th}$ ([I])	$M_{n,\,Abs}$	*Đ*
1.	L- pOEOMA	200/0/1	88	-	88 000	85 000	1.09
2.	B-6%-pOEOMA	200/12/1	86	90	87 200	86 200	1.35
3.	L-pCBMA	200/0/1	84	-	44 000	65 200	1.24
4.	B-6%-pCBMA	200/12/1	86	95	46 200	61 100	1.44

Reactions conditions: $[M]_0/[IB]_0/[I]_0/[EYH_2]_0/[CuBr_2]_0/[TPMA]_0$: 200/12/1/0.01/0.2/0.6, $[M]_0$ = [OEOMA500]/[CBMA]=300 mM, $[IB]_0$ =[SBA]= 18 mM, [I]= 1.5 mM where $([I]=[CT\text{-}ibbr12]_0$ = 0.125 mM) in 1X PBS buffer, irradiated for 50 min under green light LEDs (λ_{max} = 527 nm, 50 mW cm^{-2}), in an ambient atmosphere. Reaction volume 2.0 mL, stirring at 300 rpm, Temp. = 15^{0}C -18^{0}C. ^{a}[M] and [IB] conversion was determined by using ^{1}H NMR spectroscopy. ^{b}Theoretical molecular weight ($M_{n,Th}$)

was calculated based on conversion. [c]Absolute molecular weight ($M_{n,\,abs}$) determined by SEC in 1X DPBS coupled with multi-angle light scattering detectors (MALS).

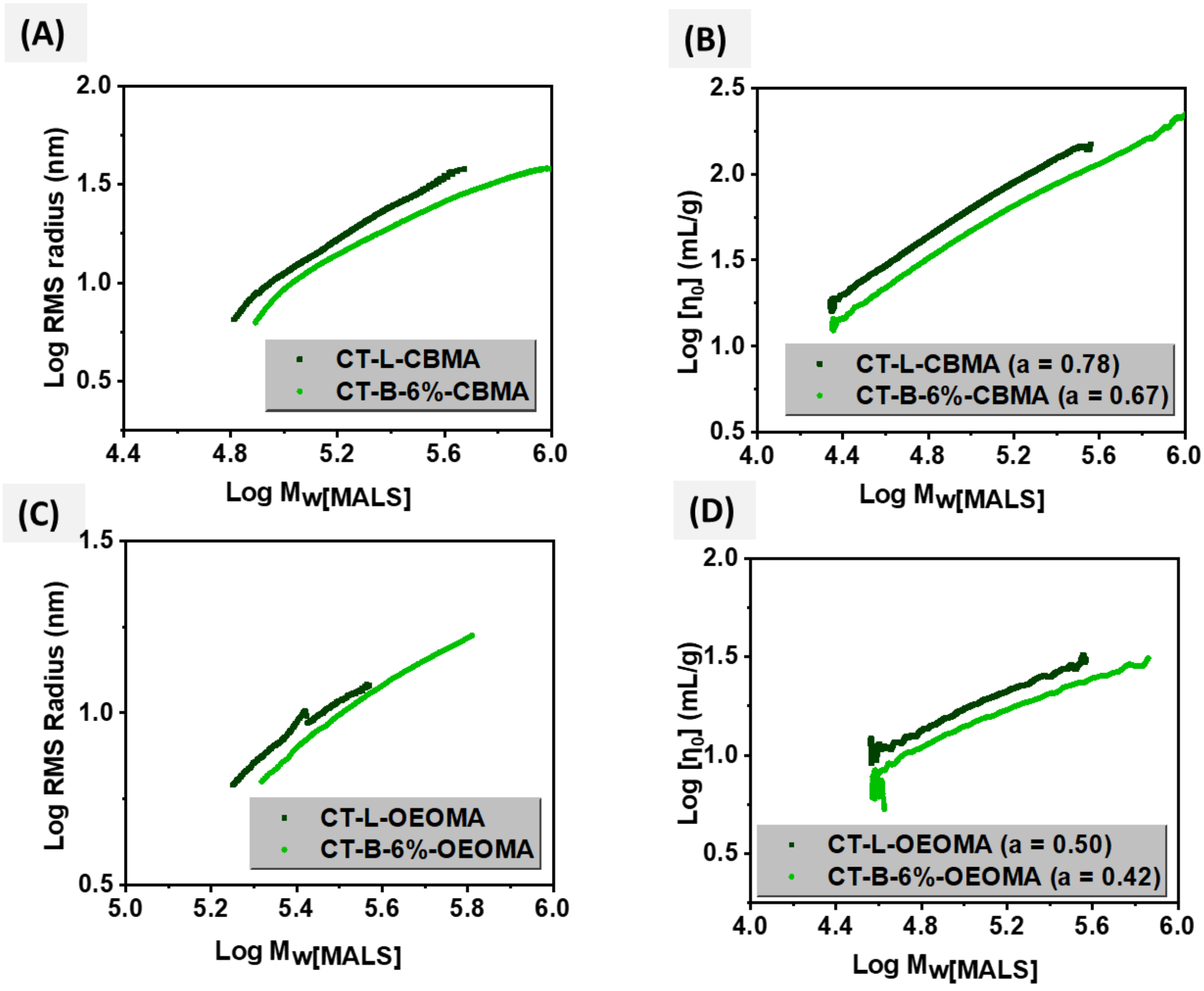

Figure S5. (A) RMS Conformation plot of L/B pCBMA (B) Mark-Houwink Sakurada (MHS) Plot L/B pCBMA (C) RMS Conformation plot of L/B pOEOMA (D) Mark-Houwink Sakurada (MHS) Plot L/B pOEOMA

The conformation polymer plots for linear pCBMA/pOEOMA and branched pCBMA/pOEOMA using both radius method (log-log plot of R versus M, Figure S5 & C) and viscosity method (log-log plot of [η] versus M, Figure S5 B & D) revealed lower slope values of branched polymers as compared to their linear counterparts of the similar molecular weights. This observation confirms the presence of branching in the polymer backbone leading to a spherical morphology and smaller hydrodynamic radius arising due to microstructural changes in the polymer architecture.

Evaluating enzymatic activity:

Methods: *N*-Succinyl-L-Ala-L-Ala-L-Pro-L-Phe-*p*-nitroanilide (suc-AAPF-pNA) was used as a substrate for enzyme bioactivity assays. In a cuvette, 0.1 M sodium phosphate buffer

(910−990 μL, pH 8.0), substrate (0−80 μL, 6 mg/mL in DMSO), and enzyme 10 μL of chymotrypsin solution in 0.1 M sodium phosphate buffer (0.1 mg/mL, pH 8.0, final CT conc. 40 nM) were mixed. The rate of the hydrolysis was determined by recording the increase in absorbance at 412 nm for the first 20 s after mixing. k_{cat}, K_M and k_{cat}/K_M values were calculated using EnzFitter software when plotting substrate concentration versus initial hydrolysis velocity (Table 2).

Table S2. Initial hydrolysis velocity of suc-AAPF-pNA by native chymotrypsin exposed under green light in polymerization conditions.

Samples		Initial velocity [a] (μM s^{-1})	Samples		Initial velocity [a] (μM s^{-1})
1	CT	1.16 ± 0.06	7	CT-L-pCBMA	0.32 ± 0.01
2	CT + green light	0.76 ± 0.03	8	CT-B-2%-pCBMA	0.48 ± 0.01
3	CT + green light + EY	0.25 ± 0.02	9	CT-B-4%pCBMA	0.56 ± 0.01
4	CT + green light + EY + L	0.33 ± 0.01	10	CT-B-6%-pCBMA	0.43 ± 0.01
5	CT + green light + EY + L +Cu	0.23 ± 0.01	11	CT-L-pOEOMA	0.08 ± 0.01
6	CT + green light + EY + L +Cu + SP	0.28 ± 0.01	12	CT-B-6%-pOEOMA	0.05 ± 0.01

[a] Initial hydrolysis velocities were estimated by CT (40 nM) with suc-AAPF-pNA (480 μM) in 0.1 M sodium phosphate buffer (pH 8.0) at 37 °C

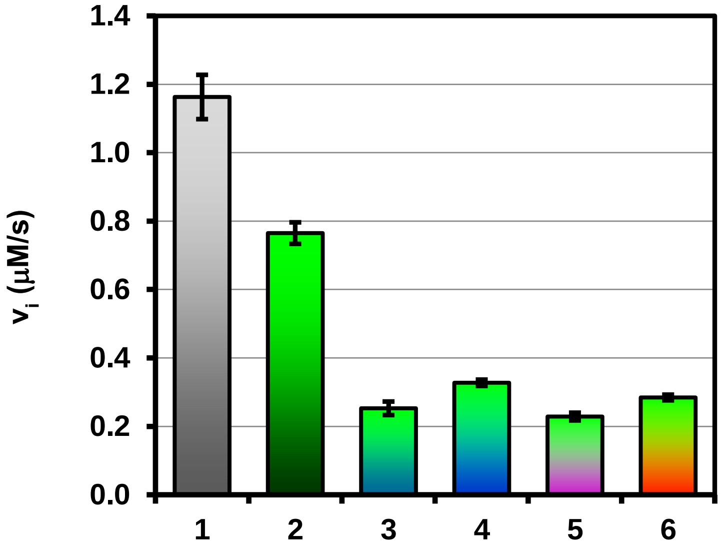

Figure S6. Comparison of the initial hydrolysis velocity of suc-AAPF-pNA by native chymotrypsin exposed under green light in polymerization conditions. 1: CT in PBS as control, 2: CT exposed by green light, 3: CT and EYH_2 exposed by green light, 4: CT, EYH_2 and ligand exposed by green light, 5: CT, EYH_2, ligand and copper exposed by green light, and 6: CT, EYH_2, ligand, copper and SP exposed by green light.

Table S3. Michaelis-Menten parameters of native CT and linear and hyperbranched polymer CT conjugates for suc-AAPF-pNA in the presence of BBI (100 nM).

Sample Name	K_M [a] (μM)	V_{max} [a] (μM s^{-1})	k_{cat} [a] (s^{-1})	k_{cat}/K_M [a] (μM^{-1} s^{-1})	Relative efficiency [b]
CT	159.1 ± 18.1	1.75 ± 0.07	43.9 ± 1.7	0.276 ± 0.034	0.596 ± 0.104
CT-L-pCBMA	84.5± 12.7	0.42 ± 0.02	11.8± 0.5	0.140 ± 0.022	0.885 ± 0.212
CT-B-2%-pCBMA	79.5 ± 8.8	0.58 ± 0.02	14.6 ± 0.4	0.183 ± 0.021	0.902 ± 0.169
CT-B-4%-pCBMA	80.7 ± 8.2	0.61 ± 0.02	15.3 ± 0.4	0.190 ± 0.020	0.766 ± 0.130
CT-B-6%-pCBMA	76.8 ± 7.9	0.47 ± 0.01	11.7 ± 0.3	0.153 ± 0.016	0.929 ± 0.154
CT-L-pOEOMA	188.5 ± 9.7	0.10 ± 0.01	2.4 ± 0.1	0.013 ± 0.001	0.891 ± 0.126
CT-B-6%-pOEOMA	171.6 ± 17.3	0.07 ± 0.01	1.7 ± 0.1	0.010 ± 0.001	0.907 ± 0.181

[a], Michaelis-Menten kinetic parameters were estimated at 37 °C for CT and conjugates with suc-AAPF-pNA. Apparent K_M and V_{max} were calculated using EnzFitter software. Apparent k_{cat} was calculated by dividing apparent V_{max} by the initial enzyme concentration, $[CT]_0$ = 40 nM.
[b], Relative efficiency is calculated from (apparent $k_{cat}/K_{M\ (BBI:\ 100nM)}$/($k_{cat}/K_{M\ (without\ BBI)}$).

Table S4. Michaelis-Menten parameters of native CT and linear and hyperbranched polymer CT conjugates for suc-AAPF-pNA in the presence of BBI (200 nM).

Sample Name	K_M [a] (μM)	V_{max} [a] (μM s^{-1})	k_{cat} [a] (s^{-1})	k_{cat}/K_M [a] (μM^{-1} s^{-1})	Relative efficiency [b]
CT	222.1 ± 18.1	1.74 ± 0.05	43.5 ± 1.3	0.196 ± 0.017	0.423 ± 0.063
CT-L-pCBMA	81.4 ± 11.5	0.38 ± 0.02	9.6 ± 0.4	0.118 ± 0.017	0.749 ± 0.175
CT-B-2%-pCBMA	90.0 ± 17.0	0.60 ± 0.03	15.0 ± 0.8	0.167 ± 0.033	0.821 ± 0.202
CT-B-4%-pCBMA	84.5 ± 13.9	0.62 ± 0.03	15.5 ± 0.7	0.183 ± 0.028	0.741 ± 0.160
CT-B-6%-pCBMA	81.6 ± 11.8	0.48 ± 0.02	12.1 ± 0.5	0.148 ± 0.022	0.902 ± 0.178
CT-L-pOEOMA	178.2 ± 28.0	0.09 ± 0.01	2.3 ± 0.1	0.013 ± 0.002	0.888 ± 0.186
CT-B-6%-pOEOMA	207.0 ± 32.7	0.08 ± 0.01	2.0 ± 0.1	0.010 ± 0.002	0.899 ± 0.212

[a], Michaelis-Menten kinetic parameters were estimated at 37 °C for CT and conjugates with suc-AAPF-pNA. Apparent K_M and V_{max} were calculated using EnzFitter software. Apparent k_{cat} was calculated by dividing apparent V_{max} by the initial enzyme concentration, $[CT]_0$ = 40 nM.
[b], Relative efficiency is calculated from (apparent $k_{cat}/K_{M\ (BBI:\ 200nM)}$/($k_{cat}/K_{M\ (without\ BBI)}$).

Table S5. Michaelis-Menten parameters of native CT and linear and hyperbranched polymer CT conjugates for suc-AAPF-pNA in the presence of BBI (400 nM).

Sample Name	K_M [a] (μM)	V_{max} [a] (μM s^{-1})	k_{cat} [a] (s^{-1})	k_{cat}/K_M [a] (μM^{-1} s^{-1})	Relative efficiency [b]
CT	477.8 ± 47.2	2.10 ± 0.11	52.5 ± 2.7	0.110 ± 0.012	0.237 ± 0.039
CT-L-pCBMA	83.1 ± 7.7	0.37 ± 0.01	9.2 ± 0.2	0.111 ± 0.011	0.702 ± 0.144
CT-B-2%-pCBMA	87.9 ±12.8	0.58 ± 0.02	14.6 ± 0.6	0.166 ± 0.025	0.818 ± 0.174
CT-B-4%-pCBMA	108.7 ± 17.6	0.65 ± 0.03	16.2 ± 0.8	0.149 ± 0.025	0.602 ± 0.130
CT-B-6%-pCBMA	100.3 ± 11.5	0.47 ± 0.02	11.8 ± 0.4	0.118 ± 0.014	0.715 ± 0.125
CT-L-pOEOMA	230.2 ± 30.6	0.10 ± 0.01	2.4 ± 0.1	0.010 ± 0.001	0.717 ± 0.137
CT-B-6%-pOEOMA	189.6 ± 29.7	0.07 ± 0.01	1.7 ± 0.1	0.009 ± 0.002	0.834 ± 0.198

[a] Michaelis-Menten kinetic parameters were estimated at 37 °C for CT and conjugates with suc-AAPF-pNA. Apparent K_M and V_{max} were calculated using EnzFitter software. Apparent k_{cat} was calculated by dividing apparent V_{max} by the initial enzyme concentration, $[CT]_0$ = 40 nM.

[b] Relative efficiency is calculated from (apparent $k_{cat}/K_{M\ (BBI:\ 400nM)}$)/($k_{cat}/K_{M\ (without\ BBI)}$).

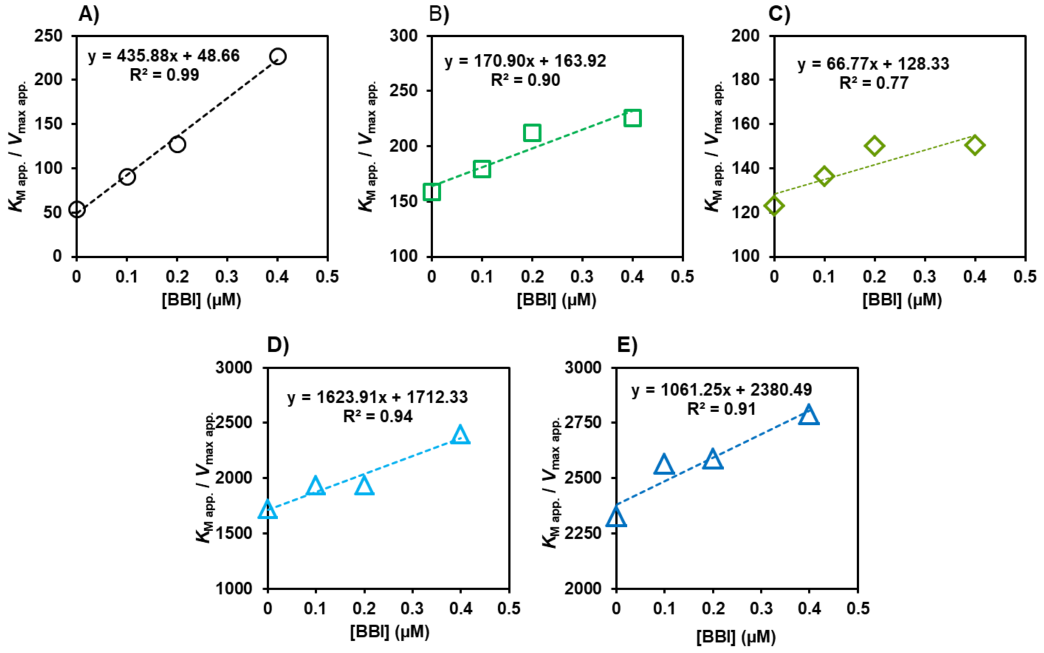

Figure S7. Secondary plots to determine inhibitor constant for native CT and CT-polymer conjugates with BBI. (A) native CT, (B) CT-L-pCBMA, (C) CT-B-2%-pCBMA, (D) CT-L-pOEOMA, and (E) CT-B-6%-pOEOMA.

Table S6. Michaelis-Menten parameters of native CT and linear and hyperbranched polymer CT conjugates for suc-AAPF-pNA in the presence of AP (100 nM).

Sample Name	K_M [a] (μM)	V_{max} [a] (μM s^{-1})	k_{cat} [a] (s^{-1})	k_{cat}/K_M [a] (μM^{-1} s^{-1})	Relative efficiency [b]
CT	214.5 ± 7.8	1.70 ± 0.02	42.6 ± 0.6	0.199 ± 0.008	0.429 ± 0.055
CT-L-pCBMA	96.0 ± 16.9	0.40 ± 0.02	9.9± 0.5	0.140 ± 0.022	0.654 ± 0.169
CT-B-2%-pCBMA	97.7 ± 10.9	0.59 ± 0.02	14.7 ± 0.5	0.183 ± 0.021	0.740 ± 0.140
CT-B-4%-pCBMA	78.0 ± 15.4	0.55 ± 0.03	13.8 ± 0.7	0.190 ± 0.020	0.714 ± 0.174
CT-B-6%-pCBMA	138.8 ± 19.9	0.57 ± 0.03	11.7 ± 0.3	0.153 ± 0.016	0.627 ± 0.124
CT-L-pOEOMA	217.7 ± 40.3	0.15 ± 0.01	2.1 ± 0.1	0.013 ± 0.001	0.660 ± 0.157
CT-B-6%-pOEOMA	206.9 ± 25.9	0.08 ± 0.01	1.6 ± 0.1	0.010 ± 0.001	0.726 ± 0.156

[a], Michaelis-Menten kinetic parameters were estimated at 37 °C for CT and conjugates with suc-AAPF-pNA. Apparent K_M and V_{max} were calculated using EnzFitter software. Apparent k_{cat} was calculated by dividing apparent V_{max} by the initial enzyme concentration, $[CT]_0$ = 40 nM.
[b], Relative efficiency is calculated from (apparent $k_{cat}/K_{M\ (AP:\ 100nM)}$)/($k_{cat}/K_{M\ (without\ AP)}$).

Table S7. Michaelis-Menten parameters of native CT and linear and hyperbranched polymer CT conjugates for suc-AAPF-pNA in the presence of AP (200 nM).

Sample Name	K_M [a] (μM)	V_{max} [a] (μM s^{-1})	k_{cat} [a] (s^{-1})	k_{cat}/K_M [a] (μM^{-1} s^{-1})	Relative efficiency [b]
CT	411.6 ± 18.4	1.93 ± 0.04	48.4 ± 1.1	0.188 ± 0.006	0.254± 0.033
CT-L-pCBMA	124.5 ± 15.5	0.43 ± 0.02	10.7 ± 0.4	0.086 ± 0.011	0.547 ± 0.122
CT-B-2%-pCBMA	142.7 ± 18.5	0.72 ± 0.03	18.0 ± 0.7	0.126 ± 0.017	0.622 ± 0.125
CT-B-4%-pCBMA	124.0 ± 15.9	0.59 ± 0.02	14.8 ± 0.6	0.119 ± 0.016	0.481 ± 0.091
CT-B-6%-pCBMA	163.1 ± 18.9	0.50 ± 0.02	12.4 ± 0.5	0.076 ± 0.009	0.464 ± 0.082
CT-L-pOEOMA	237.0 ± 26.6	0.08 ± 0.01	1.9 ± 0.1	0.008 ± 0.001	0.551 ± 0.098
CT-B-6%-pOEOMA	211.6 ± 27.0	0.06 ± 0.01	1.4 ± 0.1	0.007 ± 0.001	0.612 ± 0.133

[a], Michaelis-Menten kinetic parameters were estimated at 37 °C for CT and conjugates with suc-AAPF-pNA. Apparent K_M and V_{max} were calculated using EnzFitter software. Apparent k_{cat} was calculated by dividing apparent V_{max} by the initial enzyme concentration, $[CT]_0$ = 40 nM.

[b], Relative efficiency is calculated from (apparent $k_{cat}/K_{M\,(AP:\,200nM)}$/($k_{cat}/K_{M\,(without\,AP)}$).

Table S8. Michaelis-Menten parameters of native CT and linear and hyperbranched polymer CT conjugates for suc-AAPF-pNA in the presence of AP (400 nM).

Sample Name	K_M [a] (μM)	V_{max} [a] (μM s^{-1})	k_{cat} [a] (s^{-1})	k_{cat}/K_M [a] (μM^{-1} s^{-1})	Relative efficiency [b]
CT	773.2 ± 25.4	1.89 ± 0.07	45.7 ± 1.7	0.059 ± 0.003	0.128 ± 0.017
CT-L-pCBMA	188.5± 15.2	0.44 ± 0.01	11.0 ± 0.3	0.058 ± 0.005	0.369 ± 0.074
CT-B-2%-pCBMA	190.9 ± 24.2	0.71 ± 0.03	17.8 ± 0.8	0.093 ± 0.013	0.458 ± 0.092
CT-B-4%-pCBMA	199.8 ± 35.6	0.58 ± 0.04	14.5 ± 0.9	0.073 ± 0.014	0.293 ± 0.068
CT-B-6%-pCBMA	236.8 ± 24.7	0.49 ± 0.02	12.2 ± 0.5	0.052 ± 0.006	0.314± 0.053
CT-L-pOEOMA	271.7 ± 25.9	0.08 ± 0.01	2.0 ± 0.1	0.007 ± 0.001	0.504 ± 0.084
CT-B-6%-pOEOMA	276.8 ± 42.4	0.06 ± 0.01	1.6 ± 0.1	0.006 ± 0.001	0.521 ± 0.122

[a], Michaelis-Menten kinetic parameters were estimated at 37 °C for CT and conjugates with suc-AAPF-pNA. Apparent K_M and V_{max} were calculated using EnzFitter software. Apparent k_{cat} was calculated by dividing apparent V_{max} by the initial enzyme concentration, $[CT]_0$ = 40 nM.
[b], Relative efficiency is calculated from (apparent $k_{cat}/K_{M\,(AP:\,400nM)}$/($k_{cat}/K_{M\,(without\,AP)}$).

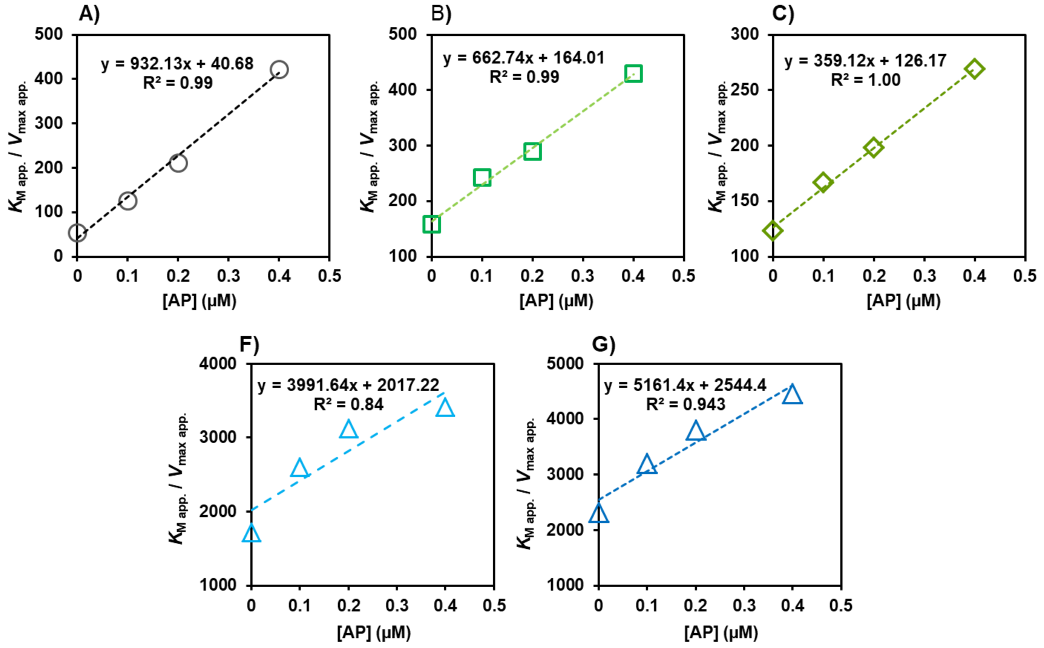

Figure S8. Secondary plots to determine inhibitor constant for native CT and CT-polymer conjugates with AP. (A) native CT, (B) CT-L-pCBMA, (C) CT-B-2%-pCBMA, (D) CT-L-pOEOMA, and (E) CT-B-6%-pOEOMA.

Table S9. Inhibition constants of the Bowman-Birk Trypsin-Chymotrypsin inhibitor from Glycine max (BBI) and Aprotinin (AP) for native CT and CT-polymer conjugates.

	K_i (nM)	
**samples**	**BBI**	**AP**
**CT nat**	**111.6**	**43.6**
**CT-L-pCBMA**	**959.2**	**247.5**
**CT-B-2%-pCBMA**	**1921.9**	**351.3**
**CT-L-pOEOMA**	**1054.4**	**505.4**
**CT-B-6%-pOEOMA**	**2243.2**	**493.0**

Table S10. Michaelis-Menten parameters and inhibitor constant of BBI of native CT exposed under green light in polymerization condition (sample 5 in Table S2).

	K_M [a]	V_{max} [a]	k_{cat} [a]	k_{cat}/K_M [a]	K_i [b]
	(μM)	(μM s^{-1})	(s^{-1})	(μM^{-1} s^{-1})	(nM)
Sample 5	80.3 ± 11.6	0.19 ± 0.01	4.7 ± 0.17	0.059 ± 0.009	29.0

[a], Michaelis-Menten kinetic parameters were estimated at 37 °C for CT and conjugates with suc-AAPF-pNA. Apparent K_M and V_{max} were calculated using EnzFitter software. Apparent k_{cat} was calculated by dividing apparent V_{max} by the initial enzyme concentration, $[CT]_0$ = 40 nM.
[b], Inhibitor constant (K_i) of BBI of native CT exposed under green light in polymerization condition was determined from slope of secondary plot with calculated apparent K_M and V_{max} values and inhibitor concentrations (Figure S8).

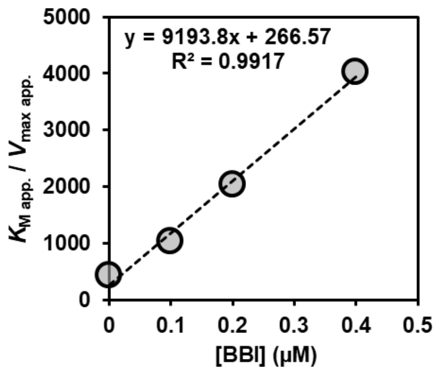

Figure S9. Secondary plots to determine inhibitor constant for native CT exposed under green light in polymerization condition (sample 5 in Table S2).

Methods:

Inhibition Assay with Bowman-Birk inhibitor (BBI) and aprotinin (AP). To assess the sensitivity of native CT and linear and hyperbranched polymer-CT conjugates inhibition by BBI and AP, the enzymatic activity of CT (40 nM) was measured with suc-AAPF-pNA (0 – 770 μM) in the presence of BBI or AP (100, 200, and 400 nM) in 0.1M sodium phosphate buffer (pH 8.0) at 37 °C for 20 s. Apparent k_{cat}, K_M and k_{cat}/K_M values in the presence of BBI or AP were calculated using EnzFitter software when plotting substrate concentration versus initial hydrolysis velocity. Activity in the presence of an inhibitor was compared to activity without an inhibitor. Concentrations of protein inhibitors were taken from previous report.[6]

Apparent K_i values for native and conjugates were estimated from secondary plots with calculated apparent K_M and V_{max} values and inhibitor concentrations using equation (2) pbelow.

$$slope = \frac{K_M}{V_{max} \cdot K_i} \qquad (1)$$

$$K_i = \frac{\frac{K_M}{V_{max}}}{slope} \qquad (2)$$

References

(1) Baker, S. L.; Murata, H.; Kaupbayeva, B.; Tasbolat, A.; Matyjaszewski, K.; Russell, A. J. Charge-Preserving Atom Transfer Radical Polymerization Initiator Rescues the Lost Function of Negatively Charged Protein-Polymer Conjugates. *Biomacromolecules* **2019,** *20,* 2392–2405.
(2) Averick, S.; Simakova, A.; Park, S.; Konkolewicz, D.; Magenau, A. J. D.; Mehl, R. A.; Matyjaszewski, K. ATRP under biologically relevant conditions: Grafting from a protein. *ACS Macro Letters* **2012**, *1*, 6-10.
(3) Murata, H.; Cummings, C. S.; Koepsel, R. R.; Russell, A. J. Polymer-Based Protein Engineering Can Rationally Tune Enzyme Activity, pH-Dependence, and Stability. *Biomacromolecules* **2013**, *14*, 1919-1926.
(4) Cummings, C.; Murata, H.; Koepsel, R.; Russell, A. J. Tailoring enzyme activity and stability using polymer-based protein engineering. *Biomaterials* **2013**, *34*, 7437-7443.
(5) Cummings, C.; Murata, H.; Koepsel, R.; Russell, A. J. Dramatically Increased pH and Temperature Stability of Chymotrypsin Using Dual Block Polymer-Based Protein Engineering. *Biomacromolecules* **2014**, *15*, 763-771.
(6) Murata, H.; Cummings, C. S.; Koepsel, R. R.; Russell, A. J. Rational Tailoring of Substrate and Inhibitor Affinity via ATRP Polymer-Based Protein Engineering. *Biomacromolecules* **2014**, *15*, 2817-2823.
